# Temperature Dependent Control of the R27 Conjugative Plasmid Genes

**DOI:** 10.3389/fmolb.2020.00124

**Published:** 2020-07-10

**Authors:** Marta Gibert, Sonia Paytubi, Cristina Madrid, Carlos Balsalobre

**Affiliations:** Department of Genetics, Microbiology and Statistics, University of Barcelona, Barcelona, Spain

**Keywords:** plasmid conjugation, temperature-dependent control, TrhR/TrhY-HtdA, transcriptional regulation, R27

## Abstract

Conjugation of R27 plasmid is thermoregulated, being promoted at 25°C and repressed at 37°C. Previous studies identified plasmid-encoded regulators, HtdA, TrhR and TrhY, that control expression of conjugation-related genes (*tra*). Moreover, the nucleoid-associated protein H-NS represses conjugation at non-permissive temperature. A transcriptomic approach has been used to characterize the effect of temperature on the expression of the 205 R27 genes. Many of the 35 *tra* genes, directly involved in plasmid-conjugation, were upregulated at 25°C. However, the majority of the non-*tra* R27 genes—many of them with unknown function—were more actively expressed at 37°C. The role of HtdA, a regulator that causes repression of the R27 conjugation by counteracting TrhR/TrhY mediated activation of *tra* genes, has been investigated. Most of the R27 genes are severely derepressed at 25°C in an *htdA* mutant, suggesting that HtdA is involved also in the repression of R27 genes other than the *tra* genes. Interestingly, the effect of *htdA* mutation was abolished at non-permissive temperature, indicating that the HtdA-TrhR/TrhY regulatory circuit mediates the environmental regulation of R27 gene expression. The role of H-NS in the proposed model is discussed.

## Introduction

Horizontal gene transfer (HGT) is a process of genetic exchange that highly contributes to evolution and adaptation of bacteria to new niches by promoting acquisition of genes coding for different metabolic pathways, toxins, adhesins, or antimicrobial resistances. Plasmid conjugation is one of the main HGT mechanisms responsible for the rapid dissemination of antibiotic resistances between pathogenic strains (Bennett, 2008; Smillie et al., 2010). In order to develop strategies to avoid multidrug spread, a detailed knowledge of the conjugation process and the mechanisms regulating that transfer process is needed.

In our research group we have focused in the study of the IncHI conjugative plasmids, which are associated with multidrug resistance in several pathogens, like *Salmonella enterica* and *Escherichia coli* (Holt et al., 2011). The conjugation of this incompatibility group plasmid is thermosensitive, showing higher conjugation frequencies at low temperatures, between 22 and 30°C (Taylor and Levine, 1980). This feature suggests that the dissemination of resistances among pathogenic species by IncHI plasmids is enhanced in water and soil environments (Maher and Taylor, 1993). In addition to temperature, the physiological state of the cell is critical to define the conjugative potential of the donor cells during the transfer process of R27 plasmid, the prototype of IncHI1 plasmids (Gibert et al., 2016). The possible role of other environmental factors in the conjugation of R27, such as osmolarity, anaerobiosis, quorum sensing and acidity, has been tested (Alonso et al., 2005). No significant influence of these environmental parameters was found on R27 transfer frequency.

The genes involved in the conjugation process of the R27 plasmid, denominated *tra* (transfer) genes, are clustered in two separated regions, Tra1 and Tra2 (Sherburne et al., 2000). Each region contains three operons: R, F and H operons in the Tra1 region (Lawley et al., 2002) and AC, Z and AN operons in the Tra2 region (Rooker et al., 1999; Lawley et al., 2003). We described a regulatory circuit, highly conserved among the IncHI plasmids, composed of three R27-encoded elements, HtdA, TrhR and TrhY, which is crucial in the temperature control of conjugation of R27 plasmid (Gibert et al., 2013, 2014). TrhR and TrhY are both required simultaneously to activate transcriptional expression of the F, H, AC and Z operons and consequently to promote R27 conjugation. HtdA has an overall negative role by counteracting the stimulatory activity of TrhR and TrhY (Gibert et al., 2013, 2014, 2016).

Most regulatory studies of R27 conjugation focused on the effect of different environmental or genetic factors on the expression of one or several *tra* genes by using gene fusions and/or mRNA quantification. In a recent report, we demonstrated that, other genetic loci located outside the *tra* regions seem to be coregulated together with the *tra* genes and, by their putative functions, might be also involved in promoting an efficient R27 conjugation (Gibert et al., 2016).

To further characterize the thermoregulation of R27 conjugation, the variations on the expression pattern of the totality of genes of the R27 plasmid at permissive (25°C) and non-permissive (37°C) temperatures was determined. Having in consideration that the HtdA-TrhR/TrhY regulatory circuit described seems to play a crucial role in the environmental control of the R27 conjugation, the transcriptomic studies have been extended to an *htdA* derivative of the R27 plasmid.

## Materials and Methods

### Bacterial Strains, Plasmids, and Growth Conditions

The strains used are MG1655 (Guyer et al., 1981) and its Δ*lac* derivative AAG1 (Aberg et al., 2008) harboring either R27 (Tc^R^, Grindley et al., 1972) or drR27 (R27*htdA*^−^ Tc^R^, Gibert et al., 2013) plasmids. Strain AAG1-F contains a fusion between the F operon promoter and the *lacZ* gene, located at the *attB* chromosomal locus (Gibert et al., 2013). Plasmid pBAD*trhRY* (Gibert et al., 2014) was used to overexpress the TrhR/TrhY proteins. Bacteria were routinely grown in LB (10 g/L NaCl, 10 g/L tryptone, 5 g/L yeast extract). For conjugation experiments strains were grown in PB medium (1.5 g/L meat extract, 1.5 g/L yeast extract, 5 g/L peptone, 1 g/L glucose, 3.5 g/L NaCl, 1.32 g/L KH_2_PO_4_, 4.82 g/L K_2_HP_4_·3H_2_O). M9 minimal media plates with the following composition: 1 x M9 salts, 0.2 % lactose, 10 μM thiamine and 1.5 % bactoagar, were used to differentiate donor from transconjugant cells in conjugation experiments, as described previously (Gibert et al., 2014). When needed, tetracycline (Tc) and arabinose were added at the concentration of 15 μg/mL and 0.02%, respectively. Unless indicated, all cultures were grown at either 25 or 37°C and under shaking (200 rpm) conditions.

### Strain Constructions

*lacZ* fusions with the intergenic regions containing putative promoter sequences were constructed (fragments a, b and c in **Figure 4B**). An intragenic region within R0009 gene was used as a negative control (fragment d in **Figure 4B**). PCR amplification of those regions was performed using primers pairs described in Table S1. Primers were designed to incorporate either *Eco*RI (forward primers) or *Bam*HI (reverse primers) restriction sites. The PCR-amplified fragments were cloned in plasmid pGEM-T and subsequently in pRS551, either in *Bam*HI or *Eco*RI-*Bam*HI sites. The resulting constructs were transferred to the *attB* chromosomal locus of the AAG1 strain using previously described protocols (Simons et al., 1987). Controls to confirm single gene fusion in the *attB* locus were performed for all fusions. All genetic constructions were confirmed by DNA sequencing.

### Conjugation Experiments

Mating experiments, using strains AGG1 R27 and MG1655 as donor and recipient strains, respectively, were performed as described previously (Gibert et al., 2014).

### β-Galactosidase Assay

β-Galactosidase assays were performed as described previously (Miller, 1992). Data are given as mean values from at least three independent experiments, plotted with standard deviations.

### Total RNA Isolation

The RNA used in microarray experiments was purified from three independent cultures grown in LB under shaking conditions. The temperature of incubation was either 25 or 37°C and samples were taken at mid logarithmic phase (log, OD_600nm_ of 0.4). The RNA was purified using an SV Total RNA Isolation System (Promega) according to the manufacturer's instructions. The RNA was DNase treated with TURBO DNAse (Ambion). After concentration, using a RNeasy Minielute Clean-up kit (Qiagen), purity and quality of the purified RNA was tested by Bionalyzer 2100 (Agilent Technologies). All samples show a RNA integrity number (RIN) over 8.0.

### Microarray Analysis

Transcriptomic analysis was performed on a custom-designed DNA microarray engineered by NimbleGen, containing two replicates of seven selected probes for each of the 205 annotated genes of the R27 plasmid (NC_002305), as previously described (Paytubi et al., 2014). In this work we compare the microarray data obtained from cultures of strains harboring R27 or drR27 plasmid, grown at 25 or 37°C to mid log phase of growth. Microarray data of R27 genes expression at 25°C have been previously described in a comparative study between logarithmic and stationary phase cultures (Gibert et al., 2016). The complete dataset has been deposited under the accession number E-MTAB-9150 at http://www.ebi.ac.uk/arrayexpress.

### Characterization of Transcripts

To describe the transcriptional organization of the AN operon, total RNA was isolated from cultures of strain AAG1(R27) grown in LB at 25°C to an OD_600nm_ of 2.0. To characterize the 5′ and 3′ end of the transcripts generated from the AN operon, circularized RNA was obtained as follows. CircRNA samples containing processed transcripts (monophosphated in the original RNA samples) were obtained after direct ligation of 10 μg of total RNA with CircLigase RNA Ligase (Epicentre). To obtain circRNA samples carrying primary transcripts (triphosphated in the original RNA samples), prior ligation, 10 μg of total RNA was treated with phosphatase (CIAP, Invitrogen) and subsequent RNA 5′ polyphosphatase (Epicentre). CircRNA samples were retrotranscribed to cDNA using the AMV RT (Promega) with specific primers of the AN operon (Table S2). cDNA sequences generated from the junction between the 5′- and 3′- ends were PCR amplified, using two subsequent rounds of PCR with the indicated primers (Table S2). The PCR products were further purified and sequenced with the primers used in the second round PCR by a Sanger approach using the BigDye™ Terminator v3.1 Cycle Sequencing Kit.

The 5′RACE was performed using the FirstChoice RLM-RACE kit (Ambion) and following manufacturer's instructions. After cDNA synthesis, two rounds of PCR were performed using the primer pairs outer primer/R1-trhU and inner primer/R2-trhU. The outer and inner primers are supplied by the manufacturer. The amplicons generated were purified and sequenced.

To determine cotranscription among the different transcripts generated from the AN operon, cDNA was obtained from total RNA samples using primers R1-0009, R1-trhP, and R1-trhU and the reverse transcriptase AMV RT (Promega). PCR amplification using the following primer pairs was used to detect cotranscription: F1-htdK/R1-0009, F1-0009/R1-trhP, and F1-trhW/R1-trhU. As control of DNA contamination, not retrotranscribed samples were used.

## Results and Discussion

### Transcription Profile of the R27 Plasmid at Permissive and Non-permissive Temperatures

**Figure 1:**
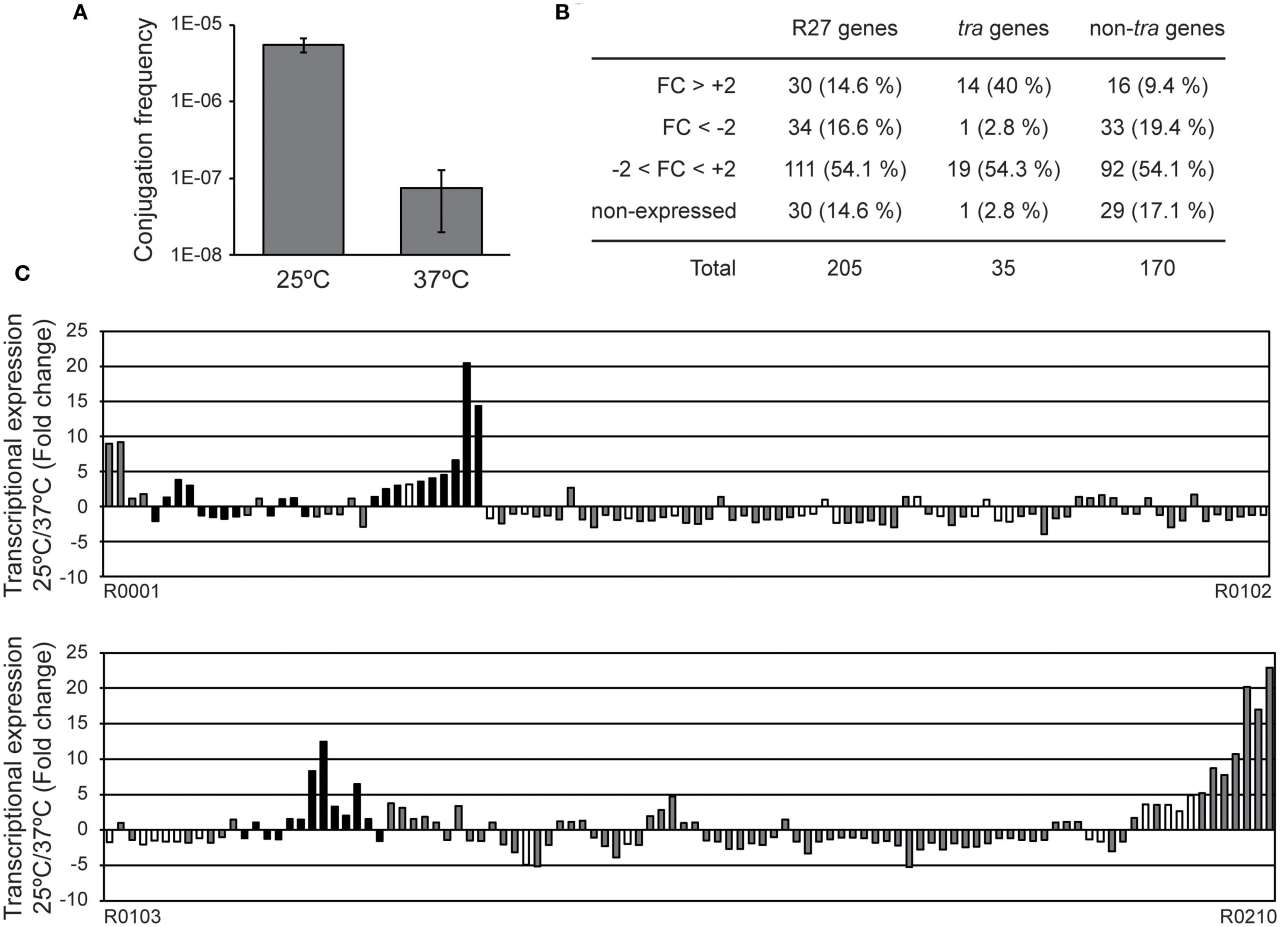
Expression profile of the R27 genes at permissive and non-permissive temperatures. **(A)** Conjugation rates were calculated using cultures of both donor [AAG1(R27)] and recipient (MG1655) strains grown at either 25 or 37°C. Average values and standard deviation of three independent experiments is shown. **(B)** Summary of the genes with an altered transcriptional expression in cultures of the strain AAG1(R27) grown at either 25 or 37°C. R27 genes are also divided in *tra* and non-*tra* genes. Attending to the fold change (FC), genes are classified in derepressed at 25°C (FC>+2), repressed at 25°C (FC < -2), no thermoregulated (-2 < FC < +2) and genes with signal values lower than 100 fluorescence units in both culture conditions were arbitrarily considered as non-expressed. **(C)** Fold change expression of the 205 ORFs encoded in R27 plasmid between AAG1(R27) cells grown at either 25 or 37°C. Expressed genes were classified as *tra* genes (black bars) and non-*tra* genes (gray bars). Non-expressed genes are indicated as white bars.

The conjugation of the R27 plasmid is tightly regulated by temperature (Figure 1A) (Taylor and Levine, 1980; Forns et al., 2005). Moreover, R27 transfer is promoted at temperatures lower than 30°C whereas is strongly repressed at 37°C. In order to characterize the effect of temperature on the expression of the R27 genes, the transcriptional expression of the 205 genes was monitored by using specific microarrays.

RNA samples extracted from AAG1 (a Δ*lac* derivative of MG1655) carrying the R27 plasmid were analyzed. Cultures were grown in LB at permissive (25°C) and non-permissive (37°C) temperatures. Previous studies showed that R27 conjugation occurs more efficiently in cells growing exponentially as compared with cells in stationary phase of growth (Gibert et al., 2016). For this reason, cultures were grown up to mid logarithmic phase (OD_600nm_ 0.4). Only expression values higher than 100 (arbitrary units of intensity of fluorescence), in at least one of the two conditions compared, were considered as significant expression and thus, the fold-change expression between conditions was calculated. We arbitrarily defined the fold-change threshold for the differential expression of a gene to 2-fold or higher. Therefore, genes with a fold-change ≥ +2 are induced at 25°C whereas genes with a fold-change ≤ −2 are induced at 37°C. A summary of the number of genes with a temperature-dependent expression (Figure 1B) and the fold change of all R27 genes (Figure 1C and Table S3) are shown. Overall, 64 genes have the expression altered by the temperature under the experimental conditions used, representing a 31.2% over the total number of R27 genes. Amongst these genes, approximately half (30) are induced at low temperature whereas the other half (34) are repressed at 25°C. No expression was detected in 30 genes, representing the 14.6% of all R27 genes. When discriminating between *tra* and non-*tra* genes, the distribution of affected genes showed different patterns. Up to 42.8% of the *tra* genes are affected by temperature. Most of them, 14 of the 15 genes affected, were stimulated at 25°C consistent with the clear increase in the conjugation ratio detected at this temperature. On the other hand, only 28.8% of the non-*tra* genes were affected, being most of them (33 of 49) repressed at 25°C. In Table 1, all genes with altered expression at low temperature are detailed. Genes from all *tra* operons, except for the Z operon coding for the entry-exclusion system, were found significantly induced at 25°C. Consistent with the increased conjugation frequency detected at low temperature, several genes from the AC operon, coding mostly for proteins required for mating pair formation, were found among the genes with the greatest induction. For instance, the gene *trhA*, coding for the major subunit of the putative conjugative pilus, is induced more than 14-fold. The expression of the R operon, coding for the regulators TrhR and TrhY, that are required for activation of *tra* operons expression, was induced at 25°C (3.2 and 1.9-fold for *trhR* and *trhY*, respectively) consistent with previous transcriptional studies (Gibert et al., 2014). Most of the non-*tra* R27 genes have not been characterized and their predicted function has been assigned attending to protein homology studies. Among the non-*tra* genes induced at 25°C, some of them might contribute to promote plasmid transfer, such as the muramidase (R0130) and a protein involved in the turnover of disulphide bonds (R0135) (Elton et al., 2005; Zahrl et al., 2005). Interestingly, all those non-*tra* genes were also found to be induced under physiological conditions promoting R27 conjugation (Gibert et al., 2016). A cluster of 9 genes, from ORF R0204 to ORF R0002, are importantly induced at low temperature. The function of the predicted proteins is not known.

**Table 1:** Expression level of the R27 genes with altered expression at either 25 or 37°C in cultures grown up to mid-logarithmic phase.

		**Gene**	**25^**°**^C**	**37^**°**^C**	**FC**	**Gene name and predicted function**
		R0007	1172	309	3.79	*trhW* (AN), pilus assembly
		R0008	299	101	2.97	*trhP* (AN), peptidase
		R0025	322	128	2.52	*trhV* (AC), lipoprotein
		R0027	528	176	2.99	*htdT* (AC)
		R0029	664	185	3.59	*trhB* (AC), Mpf formation
		R0030	558	138	4.04	*htdO* (AC)
*tra* genes	FC>+2	R0031	465	102	4.56	*trhK* (AC), pilus assembly
		R0032	1337	201	6.64	*trhE* (AC), pilus assembly
		R0033	1424	70	20.44	*trhL* (AC), pilus assembly
		R0034	1536	107	14.35	*trhA* (AC), pilus major subunit
		R0121	1207	145	8.32	(H)
		R0122	511	41	12.45	*traH* (H), relaxosome protein
		R0123	1669	510	3.27	*trhR* (R), regulator
		R0126	129	20	6.47	*trhF* (F), pilus assembly
	FC < -2	R0005	190	393	−2.06	*trhN* (AN), Mpf complex stability
		R0001	1306	147	8.91	
		R0002	172	19	9.18	
		R0042	6132	2278	2.69	partition protein
		R0129	169	45	3.76	
		R0130	183	59	3.13	*bfpH*, trbN-like protein
		R0135	1433	425	3.37	dsbA, outer membrane protein
	FC>+2	R0153	692	242	2.86	partition protein
		R0154	1714	362	4.73	cythosine methylase
		R0200	129	36	3.58	
		R0204	403	77	5.25	
		R0205	352	40	8.72	
		R0206	197	26	7.74	
		R0207	921	86	10.71	
		R0208	268	13	20.23	
		R0209	310	18	17.00	
		R0210	1201	52	22.90	
		R0023	62	181	−2.92	
		R0036	585	1422	−2.43	*repHIA*, replication protein
		R0044	42	124	−2.95	
		R0048	562	1190	−2.12	
		R0052	154	359	−2.33	
		R0053	115	282	−2.46	
non-		R0058	176	395	−2.25	
*tra* genes		R0067	98	218	−2.23	
		R0069	47	118	−2.54	
		R0070	558	1671	−3.00	
		R0075	67	177	−2.65	
		R0083	231	914	−3.95	*tetC*, transcriptional regulator
		R0094	1306	3893	−2.98	*insB1*, IS1 transposase
	FC < -2	R0095	6229	12567	−2.02	*insA*, IS1 transposase
		R0097	191	397	−2.08	*yigE*
		R0139	233	475	−2.03	
		R0140	119	378	−3.18	
		R0142	1126	5777	−5.13	
		R0143	372	779	−2.09	
		R0148	428	993	−2.32	Transposase
		R0149	58	224	−3.89	
		R0151	189	401	−2.12	
		R0159	725	1932	−2.66	
		R0160	122	325	−2.66	
		R0162	298	637	−2.13	
		R0166	259	867	−3.34	
		R0175	399	893	−2.24	
		R0176	154	807	−5.24	
		R0177	2327	6441	−2.77	*insB*, transposase
		R0180	3294	9075	−2.76	*insB*, transposase
		R0182	117	289	−2.47	*hha*, regulator
		R0183	1567	3694	−2.36	
		R0195	1684	5041	−2.99	*insD*, transposase

*The fold change of the fluorescence arbitrary units and the predicted function is indicated. The genes are divided in tra and non-tra genes. Attending to the fold change (FC), genes are classified in derepressed at 25°C (FC>+2) and repressed at 25°C (FC < -2)*.

Regarding the genes overexpressed at 37°C, only one belongs to the *tra* group of genes, *trhN*, coding for a predicted protein involved in mating pair formation complex stability (Lawley et al., 2003). The *trhN* gene was over 2-fold overexpressed at 37°C. Only a few genes with a higher expression at non-permissive temperature encoded predicted proteins with homologies to an annotated gene (Table 1). We found the *repHIA* gene (R0036), involved in plasmid replication; *tetC* (R0083), the tetracycline repressor; *insA* (R0095) and three *insB* genes (R0094, R0177 and R0180) that encode putative transposases of the insertion element IS1; the ORF R0195, encoding a IS2 transposase; and another putative transposase, R0148. Interestingly, the *hha* gene, coding for a transcriptional regulator that acts in combination with H-NS, is more expressed at 37°C, in concordance with its repressor role in the regulation of the conjugation process at non-permissive temperature described for H-NS/Hha proteins (Forns et al., 2005).

Altogether, our data clearly indicate that at 25°C there is a higher expression of the *tra* genes, consistent with the promoted R27 conjugation at low temperatures, whereas at 37°C the expression of mobile elements and transposase genes is induced. The results at high temperature might suggest that within the host (37°C), the mobilization of IS elements is induced promoting the transfer of material from the plasmid to the chromosome.

Evidently, many R27 genes were not thermoregulated. Some of R27 genes were highly expressed (higher than 600 arbitrary units of intensity of fluorescence) at both temperatures. These results could be expected, since many of these genes are involved in global processes that may take place at any temperature. Among these genes we found the tetracycline operon (*tetR, tetA*, and *tetD)*, genes involved in partitioning (*parA*/R0020 and *parB*/R0019), replication (*repHIB*) or transposition (R0046, R0076, and R0085).

### HtdA Causes Repression of R27 Genes Expression Only at Permissive Temperature

**Figure 2:**
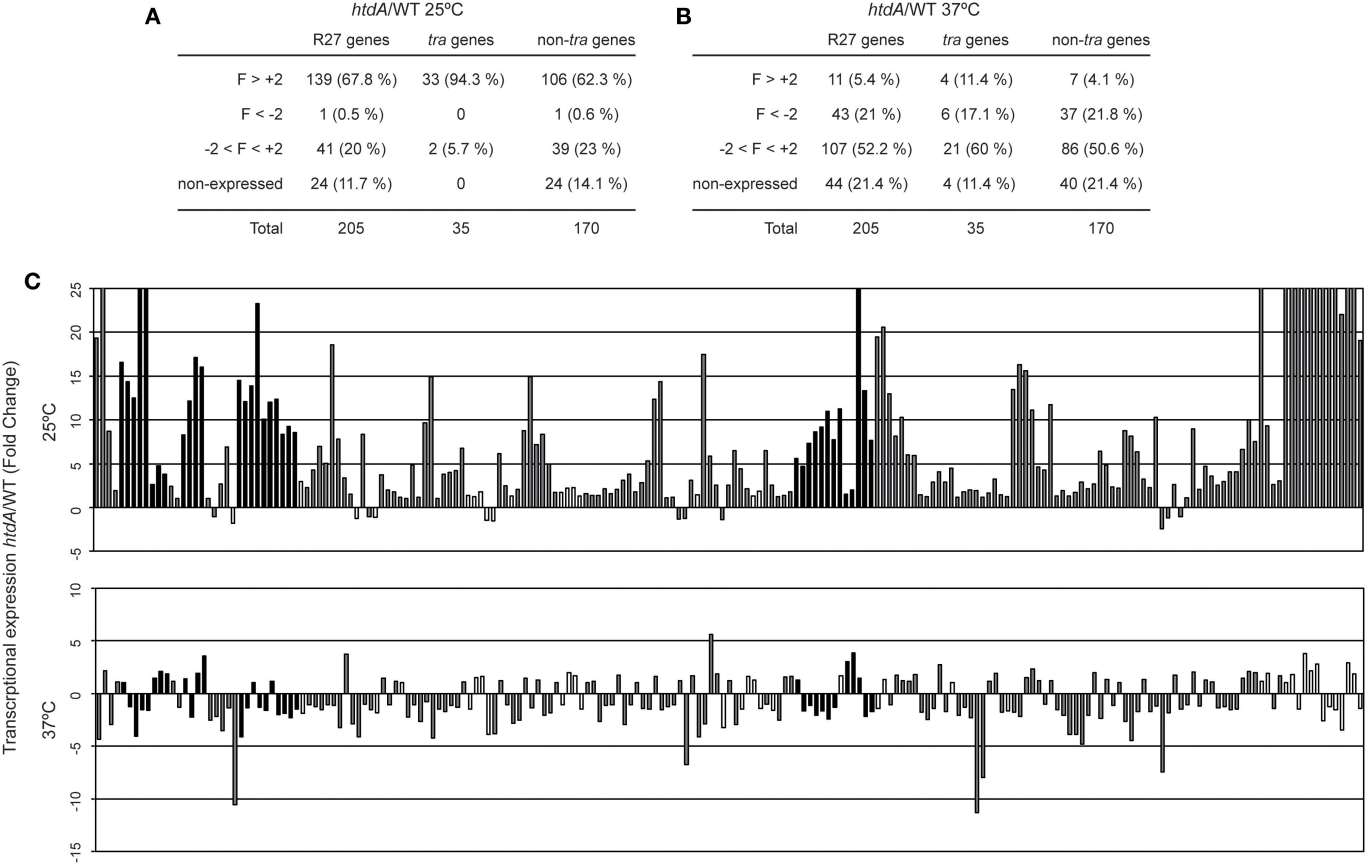
HtdA represses expression of R27 genes primarily at 25°C. Summary of the genes showing an altered transcriptional expression in cultures of the strains AAG1(drR27) and AAG1(R27) grown at either 25°C **(A)** or 37°C **(B)**. R27 genes are also divided in *tra* and non-*tra* genes. Attending to the fold change (FC), genes are classified in derepressed at (FC>+2), repressed (FC < -2), no thermoregulated (-2 < FC < +2) and genes with signal values lower than 100 fluorescence units in both culture conditions were arbitrarily considered as non-expressed. **(C)** Fold change expression of the 205 ORFs encoded in R27 plasmid between AAG1(drR27) cells and AAG1(R27) cells grown at either 25°C (upper panel) or 37°C (lower panel). Expressed genes were classified as *tra* genes (black bars) and non-*tra* genes (gray bars). Non-expressed genes are indicated as white bars.

HtdA is a R27 encoded regulator that represses conjugation since R27 plasmid transfer is strongly promoted in an *htdA* mutant (Whelan et al., 1994; Gibert et al., 2013). HtdA is involved in the repression of the transcriptional regulation of four *tra* operons (F, Z, AC, and H) by counteracting the activation mediated by TrhR/TrhY (Gibert et al., 2014). Moreover, variations in the cellular levels of HtdA seem to play a crucial role in the growth phase dependency of R27 conjugation (Gibert et al., 2016). Microarray analyses using a strain carrying a drR27 plasmid (*htdA* mutant) were performed to monitor the effect of HtdA in the transcriptional expression of the R27 genes at both permissive and non-permissive temperatures (Figure 2, Table S4). At permissive temperature (Figures 2A,C), the *htdA* mutation causes a generalized derepression of R27 plasmid genes. Under the applied experimental conditions, 139 out of 181 expressed genes were upregulated in the *htdA* mutant strain whereas only one was downregulated (R0176). The downregulated gene is a non-*tra* gene with unpredicted function. Among the *tra* genes, 33 out of the 35 genes are upregulated in the *htdA* mutant. Only the R operon, containing the *trhR* and *trhY* genes, is not upregulated more than two-fold. This is consistent with previous transcriptional data from a *lacZ* fusion with the R operon promoter, indicating that the R operon was not regulated by the HtdA protein (Gibert et al., 2013). Remarkably, TrhR and TrhY are defined as activators of the *tra* genes whose activity is counteracted at the protein level by HtdA (Gibert et al., 2014). The fact that most non-*tra* genes (106 of 170) are derepressed in an *htdA* mutant strain clearly indicates that HtdA, initially described as a regulator of the *tra* genes, controls directly or indirectly the expression of most R27 genes. Interestingly, among the genes derepressed in the *htdA* mutant, we found *hns* and *hha* which are also involved in the temperature regulation of R27 conjugation (Forns et al., 2005). An earlier study showed that H-NS silences *trhR* transcriptional expression at non-permissive temperature whereas no relevant repression was detected at 25°C (Gibert et al., 2014). The HtdA-mediated repression of H-NS/Hha at 25°C might contribute to promote expression of *trhR* and *trhY* at low temperature.

Interestingly, among the non-*tra* genes induced in the *htdA* mutant we found genes *bfph, dsbA* and two partition genes (R0042 and R00153), which have been found induced in R27 (htdA+) under all conditions known to promote R27 conjugation such as low temperature (Table S3) and log phase (Gibert et al., 2016).

Remarkably, all the 39 non-*tra* genes HtdA-independent were not induced at permissive temperature as compared to 37°C in the R27 plasmid (*htdA*^+^) (Tables S3, S4). Among these genes, 8 are involved in transposition (R0046, R0076, R0085, R0094, R0095, R0177, R0180, and R0181). We also found 2 partition genes (*parA*/R0020 and *parB*/R0019), 2 genes involved in citrate transport (*citA* and *citB*), part of the Tc operon (*tetR, tetA* and *tetC*) and 2 genes involved in UV protection (*mucA* and *mucB*) (Tables S3, S4). All these genes are involved in processes that presumably are independent of the conjugation process of the R27 plasmid.

The effect of the *htdA* mutation at non-permissive temperature was tested (Figures 2B,C) and a completely different pattern was observed. Most of the expressed genes did not show altered expression in the *htdA* mutant strain (107 of 161). Only 11 genes were derepressed by the *htdA* mutation as compared with the 139 genes detected at permissive temperature. On the other hand, at 37°C, 43 genes have lower expression in the *htdA* mutant than in the strain harboring the wt plasmid whereas at 25°C only one gene showed a decrease in the expression. Among the *tra* genes, 4 genes are derepressed in the *htdA* mutant, *htdF* from AN operon, *trhZ* from the Z operon and the 2 genes from the R operon; whereas 6 genes are repressed *trhH* (F operon), *traI* and R0118 (H operon), *trhW* (AN operon), R0016 (Z operon) and *trhC* (AC operon). Among the non-*tra* genes, only 7 genes were depressed in the *htdA* mutant, all of them were also repressed by HtdA at permissive temperature.

Overall our data suggest that the repressor role of HtdA at permissive temperature is not limited to the *tra* genes and its regulatory role vanishes at 37°C.

### Overexpression of TrhR/TrhY Induces Expression of the F Operon Even at Non-permissive Temperature

**Figure 3:**
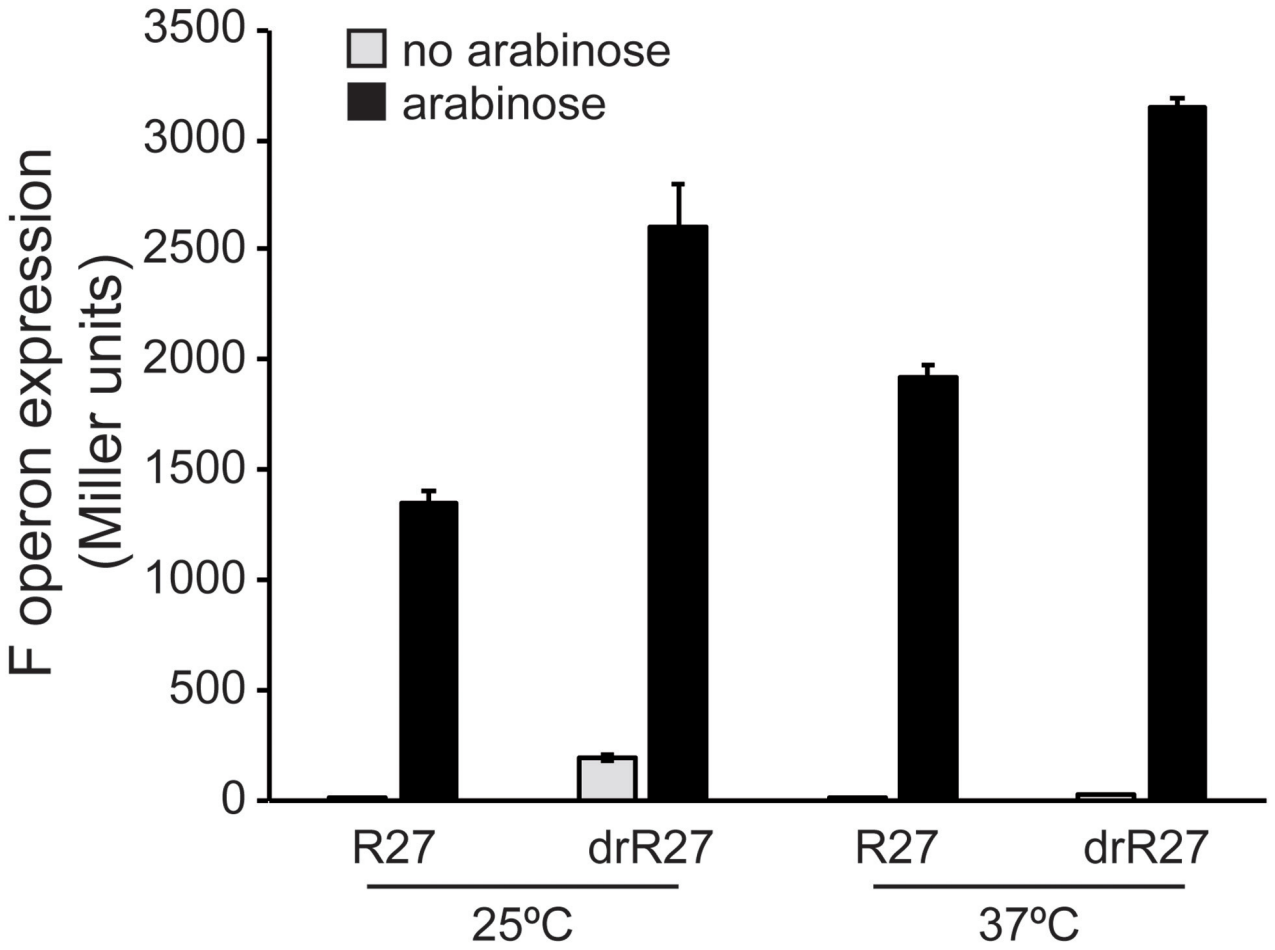
TrhR and TrhY overexpression causes induction of the F operon even at non-permissive temperature. Transcriptional expression of the F operon of R27 was monitored in cultures of the strain AAG1-F, carrying a chromosomal *lacZ* fusion with the promoter sequence of the F operon. The effect of the pBAD*trhRY* plasmid in the presence of either R27 or its *htdA* derivative drR27 was assessed. Cultures were grown at either 25 or 37°C to mid-logarithmic phase in the absence (gray bars) or presence (black bars) of arabinose (0.02%). The β-galactosidase activity (Miller units) was determined in three independent cultures and mean values with standard deviations are plotted.

Our data demonstrate a powerful repressor role of HtdA on the expression of R27 genes, especially among the *tra* genes, which is temperature dependent. Having in consideration that HtdA acts by counteracting the activation mediated by TrhR/TrhY, the role of the activators in the temperature mediated regulation was studied. F operon expression has been systematically used as a reporter to study HtdA-mediated regulation of the *tra* genes (Gibert et al., 2013, 2014, 2016). F operon is repressed by HtdA at permissive temperature but not at non-permissive temperature (Table S4). The effect of overexpressing TrhR/TrhY on F operon expression was monitored at permissive and non-permissive temperature in both *htdA*^+^ and *htdA*^−^ genetic backgrounds. Overexpression of trhR/trhY using the arabinose inducible promoter from pBAD18 causes a robust upregulation of F operon independently of the temperature (Figure 3). These results suggest that TrhR/TrhY activity is crucial for the temperature-dependent regulation of R27 conjugation and provides a rational for the mechanisms of HtdA in the thermoregulation.

### New Insights in the Transcriptional Expression of the an Operon

**Figure 4:**
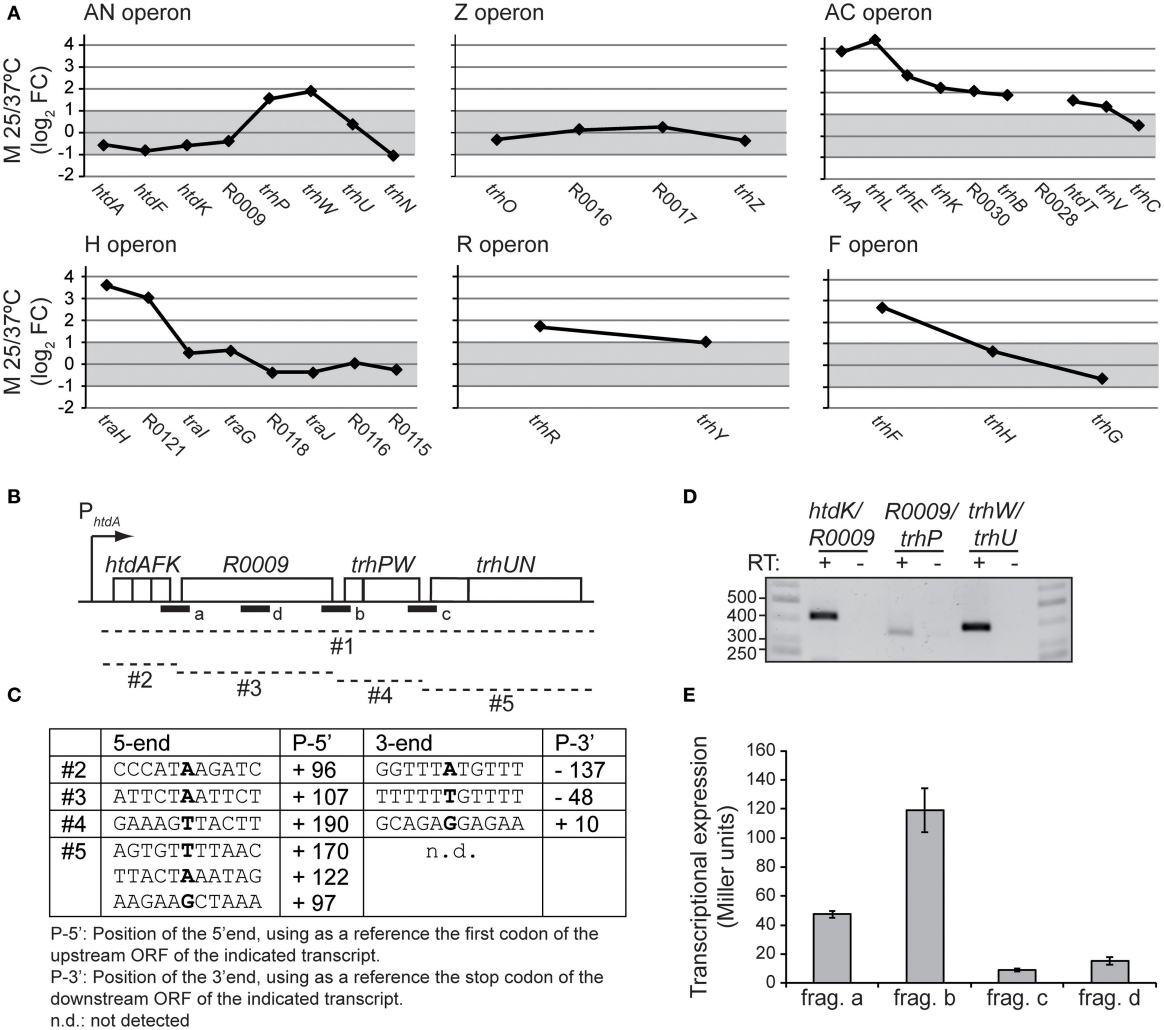
Transcriptional organization of the AN operon. **(A)** M-plots of the temperature dependent expression of the *tra* operons. The M value (log_2_FC 25/37°C) for the expressed genes of the different *tra* operons is depicted. An M value between +1 and−1, equivalent to a FC of +2 and−2, is considered no alteration in the expression and is shadowed in gray. **(B)** Representation of the AN operon. Dashed lines indicate the different putative transcripts (#1 to #5) derived from the AN operon. A thick line indicates the fragments (a to d) cloned in pRS551 to construct the *lacZ* fusions used. **(C)** The positions of 5′- and 3′ ends of the transcripts #2, #3 and #4, labeled in bold, were determined by circRNA. The putative 5′ ends of transcript #5 identified by 5′RACE assays. **(D)** RT-PCR using primers within the ORFs flanking the intergenic regions indicated. In all cases RNA not-RT AMV-treated was used as negative control. **(E)** Transcriptional expression of the *lac*Z fusions with the indicated fragments of AN operon was determined in cultures grown at 25°C to mid-logarithmic phase. The β-galactosidase activity (Miller units) was determined in three independent cultures and mean values with standard deviations are plotted.

In Figure 4A, the expression pattern of the six *tra* operons is depicted using the M value (log_2_FC) between 25 and 37°C of the different genes. Genes with no altered expression are defined by having a M value between +1 and−1, equivalent to−2 > FC > +2. A closer look to the gene expression pattern of the *tra* operon in response to the temperature reveals three different patterns. Pattern 1, unresponsiveness, as shown by the Z operon, where the expression of the three genes is not altered by temperature. Pattern 2, operons with induced expression at 25°C and the temperature responsiveness is greater among the proximal genes than among the distal ones. This is the most common pattern, shared by the operons AC, H, R, and F. The decrease in the expression of downstream genes in the same operon is defined as transcriptional polarity and it is a common feature among polycistronic operons. Pattern 3, the unusual pattern shown by the AN operon. The 4 proximal genes (*htdA, htdF, htdK* and R0009) do not respond to temperature, the 4 following genes show a pattern similar to those described in pattern 2. *trhP* and *trhW*, are induced at 25°C (2.9 and 3.7-fold) and the 2 distal genes (*trhU* and *trhN*) are again unresponsive to temperature. This expression profile within a polycistronic operon suggests the presence of complex regulatory mechanisms. Previous studies on the transcriptional organization of the *tra* genes revealed that the AN operon contains 8 genes, spanning from *htdA* to *trhN* which were apparently cotranscribed (Alonso et al., 2005). However, those studies did not rule out the occurrence of several transcripts arising from different events such as partial termination, mRNA processing and/or the possible presence of internal promoters. Interestingly, we found 3′ intergenic regions in the AN operon, located between *htdK* and R0009 (197 bp), R0009 and *trhP* (233 bp), and *trhW* and *trhU* (249 bp). Promoter search with the BPROM software (Solovyev and Salamov, 2011) predicts two putative promoters located upstream of the R009 gene and the *trhP* gene. Transcriptional terminators searches, using the ARNold software (Naville et al., 2011) identify a putative Rho-independent terminator downstream of the *htdK* gene. To characterize the transcripts generated from the AN operon, total RNA was isolated from cultures of strain AAG1(R27) grown at 25°C. Circularized RNA was obtained from either the isolated RNA (monophosphated mRNA, processed transcripts) or after 5′polyphosphatase RNA treatment (originally triphosphated, real transcriptional starts). The circRNA samples were used as template to obtain cDNA using specific primers (Table S2). Sequencing of the cDNA allowed us to clearly identify three transcripts, labeled as #2, #3 and #4 in Figures 4B,C. The fact that these three transcripts have been detected provides a feasible explanation to the particular expression profile of the AN operon independently of the mechanisms involved in its generation. Interestingly, transcript #2 (*htdA**-**htdK*) was the only transcript detected after polyphosphatase treatment, indicating a triphosphated mRNA and thus, a transcript with a real transcriptional start. Transcript #3 was detected from RNA untreated (processed transcript). Remarkably, its 5′ end overlaps with the 3′ end of transcript #2, by 46 bp. A possible explanation of this result is that transcript #3 was generated from a secondary promoter different from the promoter upstream of *htdA* and once is generated this transcript would be further processed to the final transcript #3 detected. Transcript #4 has a 5′ end located downstream of the 3′ end of transcript #3, therefore it could be generated from processing of a pre-existing polycistronic operon. After several trials, a transcript containing the sequences of the distal genes *trhU-trhN* was not identified by circRNA. However, 5′RACE experiments identified several transcripts *trhU-trhN* with distinctive 5′ ends (transcript #5). Further studies will be required to identify the exact location of the 3′ end of this transcript.

RT-PCR assays (Figure 4D) using primers from the internal sequences of the transcripts #2, #3, #4, and #5 let us detect cotranscription among the different transcripts as previously described in Alonso et al. (2005) suggesting the presence of a polycistronic transcript, named #1.

The presence of putative promoter sequences in the intergenic regions (IR) upstream of R0009, *trhP* and *trhU* was assessed. The three IR and a R0009 intragenic region used as a negative control (named a, b, c, and d in Figure 4B) were cloned in pRS551 vector, upstream of the promoterless *lacZ* gene. The *lacZ*_fusion constructs generated were transferred to the *attB* site present in the *E. coli* chromosome. Transcriptional expression was measured by a β-galactosidase assay. The results (Figure 4E) suggest the presence of putative active promoter sequences upstream the R0009 and *trhP* genes. No activity was detected in the third IR between *trhW* and *trhU*.

Further extensive experiments will be required to fully describe the transcripts generated from the AN operon and the mechanisms involved in its generation. We would like to highlight that our data identify different mRNA species that provide a feasible explanation to the differential expression from the AN operon genes as revealed by the microarray data. The results obtained suggest that different transcriptional and posttranscriptional events may participate in the control of AN operon expression, such as partial termination, processing and transcription from secondary promoters. As reported by Conway et al. (2014), a large proportion (36%) of operons in *E. coli* are complex, with internal promoters or terminators that generate multiple transcription units. For 43% of operons, differential expression of polycistronic genes was observed, despite being in the same operons, indicating that *E. coli* operon architecture allows fine-tuning of gene expression.

## Concluding Remarks

The transcriptional expression of R27 genes involved in plasmid transfer was induced at 25°C, consistent with R27 conjugation being optimal at this temperature. R27 conjugation is modulated by a regulator circuit composed by HtdA and TrhR/TrhY. Our data indicate that HtdA is not thermoregulated, while TrhR and TrhY are slightly more expressed at 25°C. Moreover, TrhR and TrhY expression is not regulated by HtdA at permissive temperature. These two proteins are responsible, at least partially, of the induction of the *tra* genes observed under these conditions (Figure 3).

**Figure 5:**
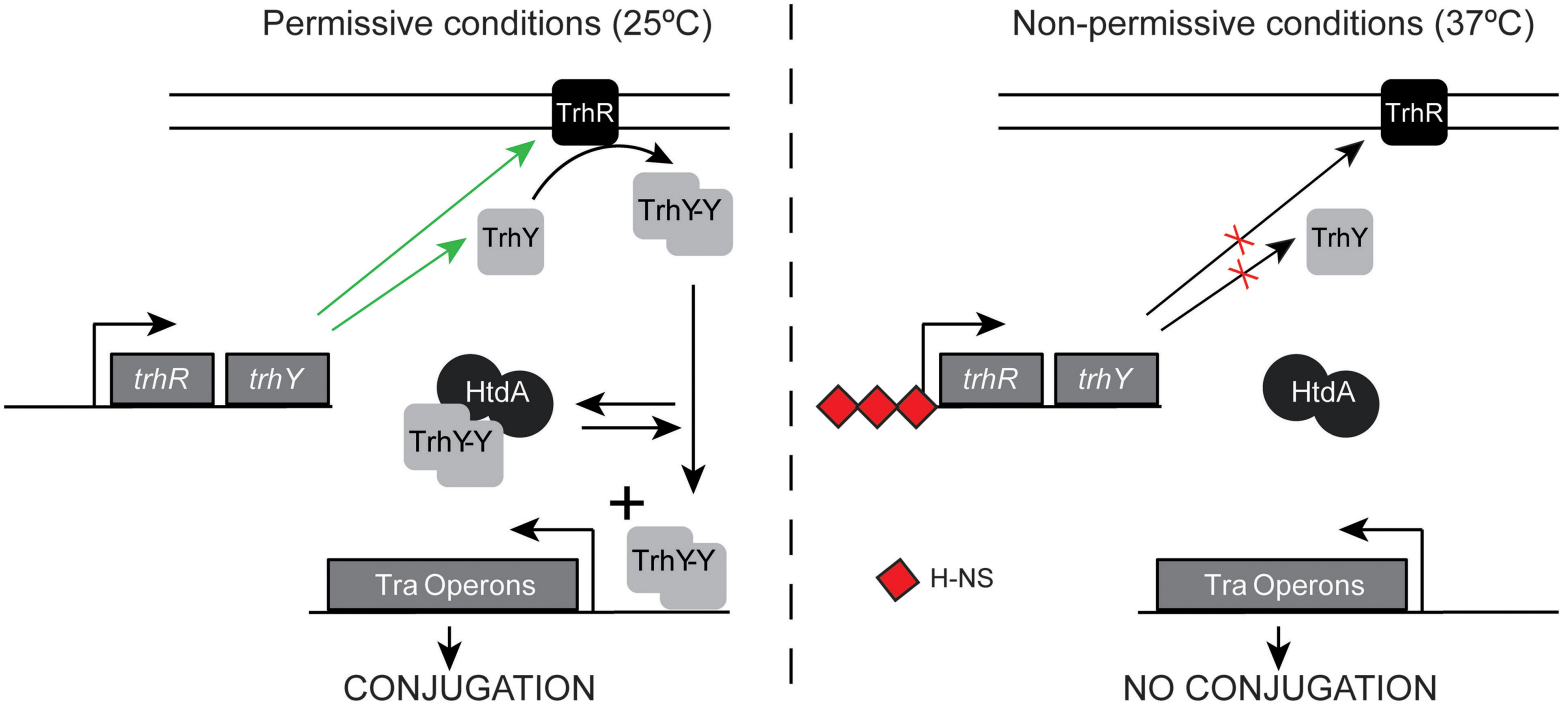
Schematic model of regulation of the *tra* operons at permissive and non-permissive temperatures. Green arrows indicate expression is promoted, whereas red crosses indicate expression is repressed.

A common expression profile shared among many *tra* and non-*tra* genes is exemplified by the *trhA* gene, coding for the pilin. A dramatic upregulation at 25°C in the absence of HtdA, but similar expression either in the presence or in the absence of the repressor at 37°C was observed (Table S4). This profile suggests that HtdA represses primarily at low temperature whereas at non-permissive temperature the presence or the absence of some specific regulators might avoid upregulation even in the absence of HtdA. HtdA acts by counteracting the activity of TrhR/TrhY and it has previously been described that H-NS silences *tra* genes expression at 37°C, by repressing *trhRY* expression (Figure 5). Having that in mind, we suggest that HtdA can exert its regulatory function only when H-NS is not blocking the expression of TrhR/TrhY. The TrhR/TrhY-HtdA mediated regulation is not restricted to the *tra* operon genes since many other R27 genes were also thermoregulated in a HtdA-dependent manner. These results also suggest that the effect of the H-NS/Hha proteins is more general as regulators of the *tra* operons than previously reported.

## Data Availability Statement

The datasets generated for this study can be found in the ArrayExpress repository, under the accession number E-MTAB-9150 (http://www.ebi.ac.uk/arrayexpress).

## Author Contributions

MG contributed in the investigation. SP contributed in the investigation and writing the manuscript. CM and CB contributed in the conceptualization, investigation, formal analysis, and writing the manuscript. All authors contributed to the article and approved the submitted version.

## Conflict of Interest

The authors declare that the research was conducted in the absence of any commercial or financial relationships that could be construed as a potential conflict of interest.
