# Corrigendum: Temperature Dependent Control of the R27 Conjugative Plasmid Genes

**DOI:** 10.3389/fmolb.2021.723430

**Published:** 2021-06-22

**Authors:** Marta Gibert, Sonia Paytubi, Cristina Madrid, Carlos Balsalobre

**Affiliations:** Department of Genetics, Microbiology and Statistics, School of Biology, Universitat de Barcelona, Barcelona, Spain

**Keywords:** plasmid conjugation, temperature-dependent control, TrhR/TrhY-HtdA, Transcriptional regulation, R27

In the original article, there was an error in the Funding statement. The funders “State Bureau of Investigation (AIE), and European Regional Development Fund (FEDER) (grant PGC 2018-096958-B-I00)” were mistakenly omitted. A correction has been made to the **Funding** statement.

“This work was supported by the Spanish Ministry of Economy and Competitiveness (grant AGL 2013-45339R), the Spanish Ministry of Science, Innovation and Universities (MCIU), State Bureau of Investigation (AIE), and European Regional Development Fund (FEDER) (grant PGC 2018-096958-B-I00), and the Catalonian government (grant 2017SGR499). MG was recipient of a FPU grant from the Spanish Ministry of Science and Innovation.”

The authors apologize for this error and state that this does not change the scientific conclusions of the article in any way. The original article has been updated.

