# Supplementary material for: Temperature Dependent Control of the R27 Conjugative Plasmid Genes
Source: Front Mol Biosci. 2020 Jul 10;7:124. doi: 10.3389/fmolb.2020.00124 (PMC7366339; doi:10.3389/fmolb.2020.00124)
Supplement: Supplementary file 1 [file Data_Sheet_1.pdf]

Supplementary Material

Temperature dependent control of the R27 conjugative plasmid genes.

Gibert, M., Paytubi, S., Madrid, C*. and Balsalobre C*

*** Correspondence:** Carlos Balsalobre: cbalsalobre@ub.edu; Cristina Madrid: cmadrid@ub.edu

Supplementary Tables

Supplementary Table S1. Primers used in the construction of *lacZ* fusions

Supplementary Table S2. Primers used in the characterization of the transcriptional organization of the AN operon by circRNA.

Supplementary Table S3. Expression level of the R27 genes at either 25ºC or 37ºC in cultures grown up to mid-logarithmic phase.

Supplementary Table S4. Expression level of the R27 genes in cultures grown up to mid-logarithmic phase of strains harboring R27 or drR27 plasmids at either 25ºC or 37ºC.

Table S1. Primers used in the construction of *lacZ* fusions. The primers amplify the intergenic regions containing putative promoter sequences (a, b and c) and the intragenic region used as a negative control (d) from the AN operon. The sequence of the restriction site introduced during PCR amplification is underlined.

**Fragment**	**Primer**	**Sequence 5'-3'**
a	K9Eco	GGGGAATTCCAGAATCGATAATTACCGCC
	K9Bam	GGGGGATCCGACGAATGCAACAAAATCGC
b	9PEco	GGGGAATTCGTTTATGGTCCGGATGAGG
	9PBam	GGGGGATCCCCAGTATCAGGATTCCGAG
c	WUEco	GGGGAATTCACGCAATTCAGTGAAGCTGA
	WUBam	GGGGGATCCATCGGGAAGATACAACTCCA
d	9Eco	GGGGAATTCCGACTTGTCGGGATTGCTTA
	9Bam	GGGGGATCCGCCCTGTAAAGCCATTTACC

Table S2. Primers used in the characterization of the transcriptional organization of the AN operon by circRNA.

**Primer**	**Sequence 5'-3'**
R1-htdA	ATCTCGACGTAAACCGTGTA
F1-htdK	TGCGTTTCAACAGGCTTGTT
R1-0009	GACGAATGCAACAAAATCGC
F1-0009	CCGTAGATTGACTTGAAACC
R1-trhP	AGTAAACCACGCTTCTTAGG
F1-trhW	ACGCAATTCAGTGAAGCTGA
R2-htdA	ATAGTTACACATGATCCCGC
F2-htdK	AAAATGCCTTGGGTATCCTC
R2-0009	ATATGAGCCTGGAGTTTTCC
F2-0009	TCGCAGTAAGGCAAGCTAAT
R2-trhP	CCTACTTCTCTCAGAATCAG
F2-trhW	ATTGGAACAAAAATCGCGGC

**TRANSCRIPT**	**cDNA primer**	**First round primer pair**	**Second round primer pair**
#2	R1-htdA	R1-htdA/F1-htdK	R2-htdA/F2-htdK
#3	R1-0009	R1-0009/F1-0009	R2-0009/F2-0009
#4	R1-trhP	R1-trhP/F1-trhW	R2-trhP/F2-trhW

Table S3. Expression level of the R27 genes at either 25ºC or 37ºC in cultures grown up to mid-logarithmic phase.

Gene	25ºC	37ºC	FC	Gene name and predicted function
R0001	1306	147	8,91	
R0002	172	19	9,18	
R0003	560	494	1,13	*trhI,* DNA helicase
R0004	9779	5433	1,80	
**R0005**	190	393	-2,06	*trhN* (AN), Mpf complex stability
**R0006**	1261	956	1,32	*trhU* (AN), Mpf complex stability
**R0007**	1172	309	3,79	*trhW* (AN), pilus assembly
**R0008**	299	101	2,97	*trhP* (AN), peptidase
**R0009**	300	390	-1,30	(AN)
**R0010**	2719	4053	-1,49	*htdK* (AN), transfer determinant
**R0011**	1045	1826	-1,75	*htdF* (AN), transfer derminant
**R0012**	738	1069	-1,45	*htdA* (AN), transfer repressor
R0013	979	1203	-1,23	ParR-like protein
R0014	5844	5020	1,16	ParM- like protein
**R0015**	487	611	-1,25	*trhO* (Z), phosphodiesterase
**R0016**	232	215	1,08	*eexB* (Z), exclusion protein
**R0017**	203	171	1,19	Lipoprotein (*Z*)
**R0018**	210	277	-1,32	*trhZ* (Z)
R0019	2418	3558	-1,47	*parB* , partition protein
R0020	2527	2625	-1,04	*parA* , partition protein
R0021	513	559	-1,09	
R0022	164	139	1,18	
R0023	62	181	-2,92	
**R0024**	271	201	1,35	*trhC* (AC), ATPase
**R0025**	322	128	2,52	*trhV* (AC), lipoprotein
**R0027**	528	176	2,99	*htdT* (AC)
**R0028**	91	29	n.e.	*htdV* (AC)
**R0029**	664	185	3,59	*trhB* (AC), Mpf formation
**R0030**	558	138	4,04	*htdO* (AC)
**R0031**	465	102	4,56	*trhK* (AC), pilus assembly
**R0032**	1337	201	6,64	*trhE* (AC), pilus assembly
**R0033**	1424	70	20,44	*trhL* (AC), pilus assembly
**R0034**	1536	107	14,35	*trhA* (AC), pilus major subunit
R0035	28	48	n.e.	
R0036	585	1422	-2,43	*repHIA* , replication protein
R0037	100	106	-1,06	
R0038	48	50	n.e.	
R0039	166	239	-1,44	zinc-finger protein
R0040	168	213	-1,27	
R0041	80	144	-1.80	DNA adenine methylase
R0042	6132	2278	2,69	partition protein
R0043	217	394	-1,81	DNA restriction methylase
R0044	42	124	-2,95	
R0045	972	1181	-1,22	
R0046	3225	6193	-1,92	*insl1,* IS30 transposase
R0047	46	78	n.e.	
R0048	562	1190	-2,12	

R0049	1691	3349	-1,98	
R0050	3411	5185	-1,52	*repHIB* , replication protein
R0051	12	15	n.e.	
R0052	154	359	-2,33	
R0053	115	282	-2,46	
R0054	99	178	-1,79	
R0055	1854	1359	1,36	
R0056	104	203	-1,95	
R0057	1573	1996	-1,27	
R0058	176	395	-2,25	
R0059	430	807	-1,88	
R0060	356	667	-1,87	
R0061	626	951	-1,52	
R0062	22	28	n.e.	
R0063	13	14	n.e.	
R0064	11	11	n.e.	
R0065	36	85	n.e.	
R0066	71	118	-1,66	
R0067	98	218	-2,23	
R0068	555	1104	-1,99	
R0069	47	118	-2,54	
R0070	558	1671	-3,00	
R0071	670	475	1,41	
R0072	82	60	n.e.	
R0073	600	637	-1,06	
R0074	52	71	n.e.	
R0075	67	177	-2,65	
R0076	1473	2120	-1,44	IS10 transposase
R0077	14	19	n.e.	*gltS* , glutamate permease
R0078	9	9	n.e.	
R0079	11	22	n.e.	
R0080	41	88	n.e.	*ydfF* , HTH regulator
R0081	4153	5738	-1,38	*tetR* , repressor protein
R0082	19056	20423	-1,07	*tetA* ,antiporter protein
R0083	231	914	-3,95	*tetC* , transcriptional regulator
R0084	638	1083	-1,70	*tetD*
R0085	1573	2306	-1,47	IS10 transposase
R0086	1392	1025	1,36	
R0087	165	131	1,25	
R0088	339	212	1,60	
R0089	3093	2523	1,23	
R0090	758	803	-1,06	
R0091	221	230	-1,04	
R0092	576	477	1,21	
R0093	302	370	-1,23	
R0094	1306	3893	-2,98	*insB1,* IS1 transposase
R0095	6229	12567	-2,02	*insA,* IS1 transposase
R0096	8688	5201	1,67	*corA,* magnesium, nickel, cobalt transporter
R0097	191	397	-2,08	*yigE*
R0098	1175	1339	-1,14	
R0099	70	135	-1.92	
R0100	168	238	-1,42	

R0101	360	429	-1,19	*flmA,* small toxic polypeptide
R0102	53	65	n.e.	
R0103	55	94	n.e.	
R0104	2196	2155	1,02	
R0105	207	295	-1,42	
R0106	20	41	n.e.	
R0107	13	19	n.e.	
R0108	22	24	n.e.	
R0109	18	30	n.e.	
R0110	160	283	-1,77	
R0111	30	36	n.e.	
R0112	92	165	-1.79	
R0113	2633	2687	-1,02	
R0114	71	102	-1.43	
**R0115**	748	881	-1,18	(H)
**R0116**	1498	1424	1,05	(H), peptidase
**R0117**	714	913	-1,28	*traJ* (H), relaxosome protein
**R0118**	806	1037	-1,29	(H)
**R0119**	459	297	1,55	*traG* (H), coupling protein
**R0120**	318	222	1,43	*traI* (H), relaxase
**R0121**	1207	145	8,32	(H)
**R0122**	511	41	12,45	*traH* (H), relaxosome protein
**R0123**	1669	510	3,27	*trhR* (R), regulator
**R0124**	397	200	1,99	*trhY* (R), regulator
**R0126**	129	20	6.45	*trhF* (F), pilus assembly
**R0127**	394	250	1,58	*trhH* (F); pilus assembly
**R0128**	354	551	-1,56	trhG (F), pilus assembly
R0129	169	45	3,76	
R0130	183	59	3,13	*bfpH,* trbN-like protein
R0131	708	464	1,53	*dsbC* , thiol:disulfide interchange protein
R0132	808	441	1,83	
R0133	387	363	1,07	
R0134	406	583	-1,44	
R0135	1433	425	3,37	*dsbA* , outer membrane protein
R0136	404	601	-1,49	*mucB,* UV protection protein
R0137	371	579	-1,56	*mucA,* UV protection protein
R0138	347	325	1,07	
R0139	233	475	-2,03	
R0140	119	378	-3,18	
R0141	10	49	n.e.	endolysin
R0142	1126	5777	-5,13	
R0143	372	779	-2,09	
R0144	623	507	1,23	*citA,* citrat-proton symport protein
R0145	650	571	1,14	*citB,* citrat utilisation protein
R0146	1035	808	1,28	*ifcA,* tricarbalyllate dehydrogenase
R0147	1461	1578	-1,08	*nac* , nitrogen assimilation regulatory protein
R0148	428	993	-2,32	transposase
R0149	58	224	-3,89	
R0150	13	26	n.e.	
R0151	189	401	-2,12	
R0152	270	140	1,93	

R0153	692	242	2,86	partition protein
R0154	1714	362	4,73	cytosine methylase
R0155	2598	2588	1,00	
R0156	2652	2527	1,05	
R0157	194	289	-1,49	
R0158	2355	3819	-1,62	*tlpA* , regulator
R0159	725	1932	-2,66	
R0160	122	325	-2,66	
R0161	430	815	-1,89	
R0162	298	637	-2,13	
R0163	71	141	-1,99	
R0164	3432	2339	1,47	*hns* , regulator
R0165	69	113	-1,63	
R0166	259	867	-3,34	
R0167	1253	2030	-1,62	
R0168	2085	2789	-1,34	
R0169	265	282	-1,06	
R0171	253	284	-1,12	
R0172	420	482	-1,15	
R0173	275	490	-1,78	
R0174	197	313	-1,59	
R0175	399	893	-2,24	
R0176	154	807	-5,24	
R0177	2327	6441	-2,77	*insB,* transposase
R0179	481	885	-1,84	*repFIB* , replication protein
R0180	3294	9075	-2,76	*insB,* transposase
R0181	6438	12337	-1,92	*insA,* transposase
R0182	117	289	-2,47	*hha* , regulator
R0183	1567	3694	-2,36	
R0184	1330	2548	-1,92	
R0185	1635	1887	-1,15	
R0186	2562	3057	-1,19	
R0187	2010	2810	-1,40	
R0188	962	1503	-1,56	
R0189	1094	1553	-1,42	
R0190	930	904	1,03	
R0191	635	540	1,18	
R0192	1380	1215	1,14	
R0193	29	39	n.e.	
R0194	12	20	n.e.	
R0195	1684	5041	-2,99	*insD,* transposase
R0197	1200	1983	-1,65	
R0198	104	61	1,70	
R0199	33	9	n.e.	
R0200	129	36	3,58	
R0201	32	9	n.e.	
R0202	27	10	n.e.	
R0203	49	10	n.e.	
R0204	403	77	5,25	
R0205	352	40	8,72	
R0206	197	26	7,74	
R0207	921	86	10,71	

R0208	268	13	20,23	
R0209	310	18	17,00	
R0210	1201	52	22,90	

The fold change and the predicted function is also indicated.
Colors indicate the genes that are higher expressed, more than two-fold, at either 25ºC (green) or 37ºC (red).
n.e. indicates not expressed (less than 100 arbitrary units) in both conditions.
Genes belonging to *tra* operons are indicated in bold.

Table S4. Expression level of the R27 genes in cultures grown up to mid-logarithmic phase of strains harboring R27 or drR27 plasmids, at either 25ºC or 37ºC.

	25°C			37°C			
Gene	drR27	R27	FC	drR27	R27	FC	Gene name and predicted function
R0001	25212	1306	19,30	34	147	-4,34	
R0002	10543	172	61,39	40	19	n.e.	
R0003	4871	560	8,70	170	494	-2,91	*trhI,* DNA helicase
R0004	18735	9779	1,92	5835	5433	1,07	
**R0005**	3158	190	16,58	407	393	1,04	*trhN* (AN), Mpf complex stability
**R0006**	18087	1261	14,34	786	956	-1,22	*trhU* (AN), Mpf complex stability
**R0007**	14643	1172	12,50	77	309	-4,03	*trhW* (AN), pilus assembly
**R0008**	8349	299	27,96	65	101	1,55	*trhP* (AN), peptidase
**R0009**	7813	300	26,09	241	390	-1,62	(AN)
**R0010**	7074	2719	2,60	5763	4053	1,42	*htdK* (AN), transfer determinant
**R0011**	4998	1045	4,78	3809	1826	2,09	*htdF* (AN), transfer derminant
**R0012**	2811	738	3,81	1984	1069	1,86	*htdA* (AN), transfer repressor
R0013	2356	979	2,41	1385	1203	1,15	ParR-like protein
R0014	5947	5844	1,02	3922	5020	-1,28	ParM- like protein
**R0015**	4018	487	8,25	855	611	1,40	*trhO* (Z), phosphodiesterase
**R0016**	2813	232	12,13	96	215	-2,25	*eexB* (Z), exclusion protein
**R0017**	3476	203	17,14	326	171	1,91	lipoprotein (*Z*)
**R0018**	3363	210	15,99	981	277	3,54	*trhZ* (Z)
R0019	2419	2418	1,00	1397	3558	-2,55	*parB*, partition protein
R0020	2433	2527	-1,04	1221	2625	-2,15	*parA*, partition protein
R0021	1378	513	2,68	158	559	-3,54	
R0022	1129	164	6,90	104	139	-1,34	
R0023	34	62	n.e.	17	181	-10,55	
**R0024**	3935	271	14,52	49	201	-4,11	*trhC* (AC), ATPase
**R0025**	3896	322	12,11	94	128	1,36	*trhV* (AC), lipoprotein
**R0027**	7343	528	13,90	186	176	1,05	*htdT* (AC)
**R0028**	2119	91	23,25	23	29	n.e.	*htdV* (AC)
**R0029**	6705	664	10,09	117	185	-1,58	*trhB* (AC), Mpf formation
**R0030**	6719	558	12,04	160	138	1,16	*htdO* (AC)
**R0031**	5753	465	12,36	52	102	1,96	*trhK* (AC), pilus assembly
**R0032**	11181	1337	8,36	108	201	-1,87	*trhE* (AC), pilus assembly
**R0033**	13214	1424	9,28	31	70	n.e.	*trhL* (AC), pilus assembly
**R0034**	13095	1536	8,53	74	107	1,44	*trhA* (AC), pilus major subunit
R0035	83	28	n.e.	26	48	n.e.	
R0036	1312	585	2,24	1308	1422	-1,09	*repHIA*, replication protein
R0037	426	100	4,24	84	106	1,26	
R0038	334	48	6,97	33	50	n.e.	
R0039	835	166	5,04	220	239	-1,08	zinc-finger protein
R0040	3113	168	18,57	193	213	-1,10	
R0041	624	80	7,77	44	144	-3,25	DNA adenine methylase
R0042	20561	6132	3,35	8514	2278	3,74	partition protein
R0043	326	217	1,50	136	394	-2,89	DNA restriction methylase
R0044	33	42	n.e.	30	124	-4,09	
R0045	8119	972	8,35	1141	1181	-1,04	
R0046	3125	3225	-1,03	4102	6193	-1,51	*insl1,* IS30 transposase
R0047	40	46	n.e.	43	78	n.e.	
R0048	2099	562	3,73	1691	1190	1,42	
R0049	3436	1691	2,03	3209	3349	-1,04	
R0050	6085	3411	1,78	6006	5185	1,16	*repHIB*, replication protein
R0051	14	12	n.e.	16	15	n.e.	
R0052	160	154	-1,04	161	359	-2,23	
R0053	556	115	4,85	269	282	-1,05	
R0054	114	99	-1,15	67	178	-2,67	
R0055	17932	1854	9,67	1747	1359	1,29	
R0056	1560	104	14,94	48	203	-4,21	
R0057	1606	1573	1,02	1360	1996	-1,47	
R0058	662	176	3,77	234	395	-1,69	
R0059	1737	430	4,04	730	807	-1,11	
R0060	1500	356	4,21	516	667	-1,29	
R0061	4252	626	6,79	1035	951	1,09	
R0062	30	22	n.e.	19	28	n.e.	
R0063	16	13	n.e.	21	14	n.e.	
R0064	19	11	n.e.	17	11	n.e.	

R0065	25	36	n.e.	22	85	n.e.	
R0066	48	71	n.e.	31	118	-3,83	
R0067	603	98	6,18	264	218	1,21	
R0068	1361	555	2,45	1024	1104	-1,08	
R0069	61	47	n.e.	42	118	-2,83	
R0070	1136	558	2,04	655	1671	-2,55	
R0071	5892	670	8,80	689	475	1,45	
R0072	1228	82	14,92	44	60	n.e.	
R0073	4318	600	7,20	805	637	1,26	
R0074	438	52	8,37	35	71	n.e.	
R0075	331	67	4,94	96	177	-1,84	
R0076	2545	1473	1,73	2198	2120	1,04	IS10 transposase
R0077	24	14	n.e.	17	19	n.e.	*gltS* , glutamate permease
R0078	19	9	n.e.	17	9	n.e.	
R0079	24	11	n.e.	37	22	n.e.	
R0080	52	41	n.e.	59	88	n.e.	*ydfF* , HTH regulator
R0081	6644	4153	1,60	5866	5738	1,02	*tetR* , repressor protein
R0082	26532	19056	1,39	23100	20423	1,13	*tetA* ,antiporter protein
R0083	313	231	1,35	343	914	-2,67	*tetC* , transcriptional regulator
R0084	1366	638	2,14	954	1083	-1,14	*tetD*
R0085	2526	1573	1,61	2217	2306	-1,04	IS10 transposase
R0086	2903	1392	2,09	1805	1025	1,76	
R0087	506	165	3,07	45	131	-2,94	
R0088	1286	339	3,79	200	212	-1,06	
R0089	5492	3093	1,78	2649	2523	1,05	
R0090	2136	758	2,82	573	803	-1,40	
R0091	1179	221	5,33	158	230	-1,46	
R0092	7103	576	12,33	767	477	1,61	
R0093	4327	302	14,35	239	370	-1,55	
R0094	1398	1306	1,07	3151	3893	-1,24	*insB1,* IS1 transposase
R0095	7235	6229	1,16	11763	12567	-1,07	*insA,* IS1 transposase
R0096	6801	8688	-1,28	6210	5201	1,19	*corA,* magnesium, nickel, cobalt transporter
R0097	156	191	-1,22	59	397	-6,71	*yigE*
R0098	3659	1175	3,11	2262	1339	1,69	
R0099	101	70	-1,44	33	135	-4,08	
R0100	2932	168	17,50	84	238	-2,85	
R0101	2111	360	5,87	2412	429	5,62	*flmA,* small toxic polypeptide
R0102	138	53	2,59	120	65	-1,84	
R0103	40	55	n.e.	29	94	n.e.	
R0104	5624	2196	2,56	2597	2155	1,21	
R0105	1338	207	6,45	101	295	-2,93	
R0106	86	20	n.e.	28	41	n.e.	
R0107	29	13	n.e.	31	19	n.e.	
R0108	29	22	n.e.	30	24	n.e.	
R0109	34	18	n.e.	21	30	n.e.	
R0110	1033	160	6,46	279	283	-1,01	
R0111	75	30	n.e.	22	36	n.e.	
R0112	116	92	-1,26	65	165	-2,55	
R0113	3638	2633	1,38	4220	2687	1,57	
R0114	126	71	-1,77	164	102	1,61	
**R0115**	4166	748	5,57	1132	881	1,28	(H)
**R0116**	7056	1498	4,71	867	1424	-1,64	(H), peptidase
**R0117**	5199	714	7,29	832	913	-1,10	*traJ* (H), relaxosome protein
**R0118**	6953	806	8,63	510	1037	-2,03	(H)
**R0119**	4227	459	9,21	179	297	-1,65	*traG* (H), coupling protein
**R0120**	3493	318	10,98	92	222	-2,41	*traI* (H), relaxase
**R0121**	9336	1207	7,73	114	145	-1,27	(H)
**R0122**	5759	511	11,28	70	41	n.e.	*traH* (H), relaxosome protein
**R0123**	2521	1669	1,51	1537	510	3,01	*trhR* (R), regulator
**R0124**	788	397	1,99	770	200	3,86	*trhY* (R), regulator
**R0126**	3523	129	27,28	28	20	n.e.	*trhF* (F), pilus assembly
**R0127**	5246	394	13,30	113	250	-2,21	*trhH* (F); pilus assembly
**R0128**	2725	354	7,69	328	551	-1,68	trhG (F), pilus assembly
R0129	3304	169	19,50	32	45	n.e.	
R0130	3770	183	20,58	79	59	n.e.	*bfpH,* trbN-like protein
R0131	9179	708	12,96	445	464	-1,04	*dsbC* , thiol:disulfide interchange protein
R0132	6558	808	8,11	771	441	1,75	
R0133	3975	387	10,27	433	363	1,19	
R0134	2428	406	5,98	665	583	1,14	

R0135	8549	1433	5,96	756	425	1,78	*dsbA* , outer membrane protein
R0136	593	404	1,47	340	601	-1,77	*mucB,* UV protection protein
R0137	469	371	1,27	236	579	-2,45	*mucA,* UV protection protein
R0138	1011	347	2,92	225	325	-1,44	
R0139	956	233	4,10	1297	475	2,73	
R0140	348	119	2,92	224	378	-1,69	
R0141	44	10	n.e.	51	49	n.e.	endolysin
R0142	1331	1126	1,18	2809	5777	-2,06	
R0143	673	372	1,81	613	779	-1,27	
R0144	1225	623	1,97	219	507	-2,32	*citA,* citrat-proton symport protein
R0145	1270	650	1,95	51	571	-11,29	*citB,* citrat utilisation protein
R0146	1209	1035	1,17	101	808	-7,98	*ifcA,* tricarbalyllate dehydrogenase
R0147	2377	1461	1,63	1797	1578	1,14	*nac* , nitrogen assimilation regulatory protein
R0148	1388	428	3,24	1884	993	1,90	transposase
R0149	83	58	n.e.	129	224	-1,74	
R0150	15	13	n.e.	16	26	n.e.	
R0151	2548	189	13,47	225	401	-1,78	
R0152	4389	270	16,27	63	140	-2,20	
R0153	10801	692	15,60	359	242	1,48	partition protein
R0154	19017	1714	11,09	837	362	2,31	cythosine methylase
R0155	12025	2598	4,63	3135	2588	1,21	
R0156	11395	2652	4,30	2478	2527	-1,02	
R0157	2280	194	11,76	358	289	1,24	
R0158	3152	2355	1,34	2467	3819	-1,55	*tlpA* , regulator
R0159	1414	725	1,95	926	1932	-2,09	
R0160	164	122	1,34	84	325	-3,89	
R0161	732	430	1,70	209	815	-3,90	
R0162	868	298	2,91	132	637	-4,82	
R0163	150	71	2,11	68	141	-2,08	
R0164	9231	3432	2,69	4671	2339	2,00	*hns* , regulator
R0165	443	69	6,44	48	113	-2,35	
R0166	1259	259	4,85	1141	867	1,32	
R0167	2946	1253	2,35	1842	2030	-1,10	
R0168	4582	2085	2,20	2832	2789	1,02	
R0169	2330	265	8,78	107	282	-2,62	
R0171	2066	253	8,17	63	284	-4,47	
R0172	2668	420	6,35	282	482	-1,71	
R0173	887	275	3,22	654	490	1,33	
R0174	449	197	2,28	185	313	-1,69	
R0175	4090	399	10,25	751	893	-1,19	
R0176	63	154	-2,45	109	807	-7,42	
R0177	2001	2327	1,16	3509	6441	-1,84	*insB,* transposase
R0179	1262	481	2,62	1564	885	1,77	*repFIB* , replication protein
R0180	3142	3294	-1,05	6264	9075	-1,45	*insB,* transposase
R0181	7261	6438	1,13	11656	12337	-1,06	*insA,* transposase
R0182	1051	117	8,96	583	289	2,02	*hha* , regulator
R0183	3231	1567	2,06	3102	3694	-1,19	
R0184	6269	1330	4,71	3270	2548	1,28	
R0185	5835	1635	3,57	2127	1887	1,13	
R0186	6528	2562	2,55	2262	3057	-1,35	
R0187	5908	2010	2,94	2302	2810	-1,22	
R0188	3894	962	4,05	985	1503	-1,53	
R0189	4461	1094	4,08	1040	1553	-1,49	
R0190	6149	930	6,61	1309	904	1,45	
R0191	6369	635	10,03	1115	540	2,07	
R0192	10360	1380	7,51	2412	1215	1,98	
R0193	1063	29	36,93	45	39	n.e.	
R0194	116	12	9,30	38	20	n.e.	
R0195	4366	1684	2,59	3511	5041	-1,44	*insD,* transposase
R0197	3609	1200	3,01	3328	1983	1,68	
R0198	3955	104	38,13	64	61	n.e.	
R0199	3245	33	97,38	16	9	n.e.	
R0200	6669	129	51,63	25	36	n.e.	
R0201	3378	32	106,71	35	9	n.e.	
R0202	2740	27	102,07	21	10	n.e.	
R0203	5679	49	115,51	28	10	n.e.	
R0204	15984	403	39,70	29	77	n.e.	
R0205	12397	352	35,17	33	40	n.e.	
R0206	9840	197	49,83	17	26	n.e.	

R0207	20305	921	22,05	25	86	n.e.	
R0208	14240	268	53,17	38	13	n.e.	
R0209	12237	310	39,52	34	18	n.e.	
R0210	22878	1201	19,05	37	52	n.e.	

The fold change and the predicted function is also indicated.
Colors indicate the genes that are higher expressed, more than two-fold, at either 25ºC (green) or 37ºC (red).
n.e. indicates not expressed (less than 100 arbitrary units) in both conditions.
Genes belonging to *tra* operons are indicated in bold.
